# Bringing together clinical and physical sciences to deliver translational ageing research: the UK experience

**DOI:** 10.1186/s12919-025-00354-0

**Published:** 2025-12-22

**Authors:** Lewis Steell, Chloe Hinchliffe, Rachel Cooper, Miles D Witham

**Affiliations:** 1 AGE Research Group, Faculty of Medical Sciences, Translational and Clinical Research Institute, Newcastle University, Newcastle Upon Tyne, UK; 2 NIHR Newcastle Biomedical Research Centre, Newcastle upon Tyne Hospitals NHS Foundation Trust, Cumbria, Northumberland, Tyne and Wear NHS Foundation Trust and Newcastle University, Newcastle Upon Tyne, Northumberland UK; 3 Translational and Clinical Research Institute, Faculty of Medical Sciences, Newcastle University, Newcastle Upon Tyne, UK

## Background

Successfully engaging with the challenges arising from population ageing will require novel healthcare strategies designed to meet the complex needs of older people [1]. This is difficult as ageing is a heterogeneous, multi-system process, and the current research and clinical care paradigm (which focuses on a single condition or organ system) is not adequate to understand, prevent and treat the dysfunction across multiple body systems that is characteristic of ageing [2]. Taking an integrative approach to research ageing across multiple systems will require novel strategies and an increased focus on interdisciplinary collaboration.

There have been major advances in our understanding of the fundamental biology of ageing (e.g. hallmarks of ageing [3]) over the last two decades, yet these advances have often failed to translate into clinical or societal benefit due to shortcomings in the translational research pathway [4]. Improving how we design and deliver translational ageing research means combining the best insights from multiple disciplines, developing and using new tools, and finding new ways to tackle the complexity inherent in ageing research. Close collaboration between biomedical and physical sciences has yielded crucial advances in knowledge and tools for both research and clinical practice, and the application of these will be essential to address the challenge of delivering effective translational ageing research.

The Physics of Medicine Network (www.physicsoflife.org.uk/physics-of-medicine) and the ART (Ageing Research Translation) of Healthy Ageing Network (www.artofhealthyageing.net) co-convened a one-day workshop in February 2024, bringing together physical, computational, biomedical, and clinician scientists across academia and industry to build interdisciplinary research capacity, and to develop novel strategies and collaborations in translational ageing research. Presentations showcased recent advances in translational ageing research, and subsequent discussions highlighted key challenges that we need to overcome and outlined important next steps towards building effective interdisciplinary collaborations. The workshop comprised four sessions: meeting the challenges of ageing research through physical sciences; medical imaging for the novel characterisation of ageing phenotypes; diagnostics and measurement; and connecting and collaborating.

## Session 1: meeting the challenges of ageing research through physical sciences

The first session summarised the challenges of individual and societal ageing on health and wellbeing and showcased the integration of physical sciences with clinical science and other disciplines to solve these challenges. To set the scene, Professor Miles Witham discussed the clinical need for translational ageing research and key considerations for the successful delivery of innovative and impactful research in this area. The current paradigm of single mechanism/organ/disease-focused clinical research fails to account for the lived realities of many older adults, who live with multiple long-term conditions (MLTC) and attendant polypharmacy [5]. Thus the majority of older adults are neglected by the siloed single-disease approach and are often excluded from research due to their clinical complexity – leading to a lack of robust evidence to inform their clinical care [6]. Effective research cannot focus on biological mechanisms in isolation without consideration of how these interact with other factors across the spectrum of lived experience. Instead, we must develop an interdisciplinary translational ageing research pathway that advances novel ideas and solutions at the intersections of expertise.

The contributions that physical sciences can make to our understanding of the fundamental biology of ageing were highlighted in two further talks. Professor Aneta Stefanovska presented on the potential utility of mathematical and physical science frameworks for studying living systems. Using mathematical and physical science frameworks to model and investigate living systems is challenging as the physical properties that govern living systems are complex and only partly understood. Living systems can be considered large networks of non-autonomous, time-varying, oscillatory processes, interacting across levels of complexity from systemic to cellular level. Professor Stefanovska introduced a novel class of physical systems, termed *Chronotaxic* systems (from *chronos* – time, and *taxis* – order), that may be ideal candidates for studying the properties of biological oscillators within living systems. Studying biological oscillators within the framework of chronotaxic systems provides a novel approach to understanding how the interactions of oscillatory biological processes vary across health, disease, and ageing. For instance, in ageing, the strength of and mutual interactions between biological oscillators become chronically altered, potentially reflecting age-related maladaptation and functional decline [7].

Dr Kevin Chalut highlighted the role of physical sciences in improving our understanding of the cellular biology of ageing through the exploration of extracellular matrix (ECM) mechanics. This work investigates the effect of altered biomechanics of the extracellular niche on cellular function, for instance on the maintenance or loss of adult stem cell and progenitor cell regenerative capacity. Using artificially developed ECM, investigators can precisely manipulate the mechanical properties of the cellular niche while also controlling the external physical environment, assessing their relative contributions to cellular ageing. Preliminary data suggest both intrinsic and extrinsic mechanical properties of ECM alter cellular function and may drive cellular ageing, with potential implications for the development of cell-based therapies, if these findings are validated in other ageing pathways in humans [8].

## Session 2: novel characterisation of ageing phenotypes: the role of imaging

Medical imaging is an excellent example of what can be achieved via interdisciplinary collaboration between physical and clinical scientists and has revolutionised clinical practice. The second session showcased how novel imaging techniques can advance understanding of age-related conditions and highlighted how innovation in imaging can translate to innovation in clinical practice. Professors Andrew Blamire and Susan Francis discussed technological advances in magnetic resonance imaging (MRI) and spectroscopy (MRS), highlighting the potential of novel imaging methods to advance understanding of age-related conditions and their underpinning mechanisms. As in wider medicine, siloed working has been the norm in imaging, which has often been single organ or tissue focused. This is despite multiparametric MRI and MRS being able to provide comprehensive assessment of whole-body, multi-organ physiology from a single series of scans, with enormous potential for the evaluation of system-wide, age-related changes in morphology and function. Harnessing the potential of imaging as a tool in translational ageing research requires a more holistic and interdisciplinary approach, bringing together expertise from different clinical specialties and medical physics. Combining imaging with genetic data or other technologies (e.g. -omics data) could further advance our ability to deeply phenotype individuals across the lifespan, providing new insights into the ageing process [9].

The benefits of interdisciplinarity were further highlighted in a presentation showcasing work from Dr Matthew Birkbeck who developed a novel method of assessing motor unit activity using MRI as part of his PhD [10]. This work built on the observation by MRI physicists and neurophysiology colleagues of artefacts in MRI muscle images that appeared to approximate the size and shape of a motor unit. This led to further investigation and the subsequent development of motor unit MRI assessment (MUMRI). Early studies using this technique have already demonstrated age-related changes in muscle and could be of diagnostic relevance in neuromuscular disease [11].

## Session 3: diagnostics and measurement

The third session showcased how physical sciences can enable measurement of real-world patient outcomes and discussed the process of turning these scientific advances into validated diagnostic tests ready for market. Professor Muhammad Imran highlighted how advances in physics and engineering now enable the use of laser and radiofrequency-based technologies to monitor people’s physiology and activity remotely in homes, care homes, and hospitals [12]. Time-resolved optical techniques can analyse the scatter of photons reflected from the chest wall to reconstruct heart sounds and breathing rate with less privacy risk than image-based techniques. WiFi based techniques use radiofrequency fields optimised for internet use that are already ubiquitous in many buildings. Human movement distorts these signals, and machine learning allows the capture of activities such as new instances of walking, sitting, and standing. Current research is exploring the use of this technology for falls prediction and AI-enabled contactless sensing.

Many people with long-term conditions experience fatigue. Current assessments of fatigue using questionnaires and self-completed diaries are prone to recall bias, subjectivity, and involve substantial patient burden. Dr Chloe Hinchliffe discussed her postdoctoral work on digital wearables as a potential solution to improve assessment and monitoring of fatigue. Digital wearables can overcome limitations of self-reported assessment since they are objective, passive, continuous, and capture real-world data. Using validated algorithms, aspects of gait (walking) can be identified from accelerometers, including step count, length of walking bouts, and gait domains such as pace, rhythm, and variability. Counter-intuitively, people who report higher levels of fatigue do not appear to be less active (based on measuring of step count and walking time) [13], suggesting that more nuanced measures may be required to fully reflect the lived experience of people experiencing fatigue.

Professor John Simpson presented a history of Newcastle’s NIHR HealthTech Research Centre (HRC), one of 14 NIHR-funded HRCs in England, known as the MedTech and in Vitro Diagnostics Co-operatives (MICs) until 2024. The HRCs have a key role in facilitating diagnostic and HealthTech evaluation, partnering with academics and the commercial sector. HRCs bring specific methodological expertise in care pathway mapping, health economics, patient and public involvement, and selection of the best study designs across the diagnostic evaluation journey. The Newcastle HRC has themes specialising in ageing and multiple long-term conditions, infection, and precision medicine with a focus on artificial intelligence and digital technology. Close and early collaborations between academics, clinicians, methodologists, and commercial partners are essential if advances in science and engineering are to translate into robustly evaluated, marketable products that meet genuine clinical needs. HRCs provide the combination of expertise needed to help good ideas become good diagnostic and HealthTech products.

## Session 4: connecting and collaborating

The final session brought together funders and representatives from networks relevant to translational ageing research to share perspectives on future funding and capacity building. Sadhana Sharma from the UK Research and Innovation (UKRI) Biotechnology and Biological Sciences Research Council (BBSRC) discussed new initiatives and funding streams from UKRI to promote interdisciplinary collaboration, with a view to supporting research that does not align with the remit of single research council. A major barrier for interdisciplinary research is finding a ‘home’ research council for funding. Therefore, UKRI have implemented schemes designed for this: the Cross Research Council Responsive Mode Pilot Scheme, the Future Leaders Fellowships, and Physics of Life. Dr Sharma also highlighted the need to improve population health, as well as focusing on equality, ageing throughout life, digital health, and mental health.

Professor Mark Leake, Chair of the Physics of Life Network (PoLNET), presented a roadmap for interdisciplinary networking through PoLNET’s aims to identify disruptive strengths and opportunities, and to assess training needs in the UK. Professor Leake highlighted the importance of building networks across disciplines, which is a key aim of the Physics of Life programme alongside its role in identifying unmet research needs and shaping key research questions. PoLNET meets these aims through a range of actions, such as an email list which includes numerous partners and funders to help advertise positions.

Professor Chris Ponting represented X-Net, a UKRI Medical Research Council funded training network providing support for researchers working at the interface between computational, mathematical sciences, and biomedical research. ​ Professor Ponting highlighted stark findings from a survey of members, reporting that over 60% of respondents experienced a negative environment when crossing disciplines. Several barriers to cross-discipline research were noted, including differences in research culture, inadequate training, time, funding, and colleague hostility. Three main areas for improvement were highlighted: increasing mobility across disciplines and sectors through specific fellowships, training, and championing of good practice; creating nurturing environments through funding, collaborations, and training; and promoting equitable evaluation via changes to funding and training structures that recognise the added complexity of interdisciplinary research. These findings have now been published in a report and are being used to advocate for positive change [14].

Professor Ilaria Bellantuono, from UKAgeNet, which represents centres of ageing research across the UK, highlighted that increased lifespan in the UK has not been accompanied by improved healthspan [15]. To meet this challenge, Professor Bellantuono championed the importance of knowledge exchange between policy makers and ageing researchers, identifying priority areas for policy makers and connecting with key stakeholders in industry and government. UKAgeNet is well placed to meet this need, with its strong interdisciplinary membership bringing together biologists, social scientists, physical scientists and clinicians with a focus on ageing. A common theme across the day, reinforced in this final session, was the value and necessity of working across disciplinary boundaries.

## Summary and reflections

This workshop was attended by approximately 60 researchers from 18 institutions representing multiple academic disciplines and clinical specialties and showcased ways that physical sciences already contribute to translational ageing research. It also highlighted ways in which enhanced interdisciplinary collaboration between physical and clinical sciences may lead to further advances in ageing research with consequent improvements in the health and wellbeing of older adults. Physical sciences are driving progress in diagnostics, imaging, and health monitoring technologies that could have benefits for clinical practice if properly supported and implemented within an effective translational research pathway.

Interdisciplinary research in ageing is challenging but essential and some barriers to successful collaboration were highlighted. Consensus emerged that communication across disciplinary boundaries (e.g. between physical and clinical sciences) is not always easy, resulting in difficulties harnessing sustained cross-disciplinary engagement from the early stages of research development. More opportunities for networking and collaboration across disciplines, with events such as this workshop, are welcomed to address this issue and enable better interdisciplinary research engagement. Addressing differences in research cultures and models of working across disciplines also requires investment in, and willingness to develop, new approaches that harness the expertise of all involved, including older people and clinicians with expertise in the care of older people. In order to develop effective collaborations involvement needs to start early – we all need to take the time to learn each other’s languages. Finally, attendees championed the value of greater interdisciplinarity for developing research capacity in translational ageing research. Promoting interdisciplinarity among early career researchers (ECR) through targeted funding streams, facilitating their ability to bridge disciplines with dedicated resources for training, was suggested as a potential pathway for accelerating the scope and success of interdisciplinary research. The workshop was itself an exemplar for just this type of skills training for ECRs, who were involved in the design of the day, presented in several of the sessions, and co-wrote this meeting report. Thankfully, interdisciplinary research funding opportunities are becoming more widely available, yet there is still a requirement to address training needs within grants for those pivoting to other areas of research.

Feedback from attendees suggested that this workshop fulfilled a valuable role in catalysing interdisciplinary conversations and learning across disciplinary boundaries. The event succeeded in providing a forum for discussion on ways to progress and an opportunity to learn each other’s languages and approaches – key aims for both the ART of Healthy Ageing Network and the Physics of Life Network. It is to be hoped that the event will lead to new collaborations and inclusion of physical sciences in translational ageing research, building on attendees shared motivation to work together towards better health and care for older adults.
